# Supplementary material for: Genetic correlations between pain phenotypes and depression and neuroticism
Source: Eur J Hum Genet. 2019 Oct 29;28(3):358–66. doi: 10.1038/s41431-019-0530-2 (PMC7028719; doi:10.1038/s41431-019-0530-2)
Supplement: Supplementary file 1 — Supplementary Table 1 [file 41431_2019_530_MOESM1_ESM.docx]

**Supplementary Table 1.** Clinical characteristics of pain phenotypes, controls and non-responders in the UK Biobank

|  | UK Biobank |  |  |  |  |  |
| --- | --- | --- | --- | --- | --- | --- |
|  | Sex (male:female) | *P* | Age | *P* | BMI(kg/m2) | *P* |
| Headache | 27,350:47,111 | < 0.001 | 54.38 (7.95) | < 0.001 | 27.5 (5.05) | < 0.001 |
| Facial pain | 879:1731 | < 0.001 | 57.3 (7.81) | 0.028 | 27.5 (5.18) | < 0.001 |
| Neck or shoulder pain | 28093:25901 | 0.70 | 57.7 (7.82) | < 0.001 | 27.8 (4.85) | < 0.001 |
| Stomach or abdominal pain | 3581:4636 | < 0.001 | 55.4 (8.30) | < 0.001 | 27.0 (4.82) | < 0.001 |
| Back pain | 23244:20747 | < 0.001 | 57.4 (8.01) | < 0.001 | 27.9 (4.87) | < 0.001 |
| Hip pain | 4119:5997 | < 0.001 | 59.3 (7.26) | < 0.001 | 28.1 (4.99) | < 0.001 |
| Knee pain | 12062:10142 | < 0.001 | 58.3 (7.64) | < 0.001 | 28.6 (4.88) | < 0.001 |
| Pain all over body | 2171:3499 | < 0.001 | 57.3 (7.59) | 0.002 | 29.6 (5.96) | < 0.001 |
| Common Controls | 71,480:77,832 | --- | 56.9 (7.97) | --- | 26.7 (4.30) | --- |
| Non-responders | 362:481 | 0.005 | 58.6 (8.15) | < 0.001 | 28.3 (5.20) | < 0.001 |

BMI: body mass index

A chi-square test was used to test the difference of gender frequency between cases and controls and an independent t test was used for other covariates.

Continuous covariates were presented as mean (standard deviation).
