# Supplementary material for: Genetic correlations between pain phenotypes and depression and neuroticism
Source: Eur J Hum Genet. 2019 Oct 29;28(3):358–66. doi: 10.1038/s41431-019-0530-2 (PMC7028719; doi:10.1038/s41431-019-0530-2)
Supplement: Supplementary file 2 — Supplementary Table 2 [file 41431_2019_530_MOESM2_ESM.docx]

**Supplementary Table 2.** The genetic correlations between eight pain phenotypes and all available psychiatric and personality traits in the LD hub.

| Traits | Headache  rg (*P)* | Facial pain  rg *(P)* |  | Neck and shoulder pain  rg (*P)* | Stomach and abdominal pain  rg *(P)* | Back pain  *rg (P)* | Hip pain  rg (*P*) | Knee pain  rg (*P)* |  | Pain all over body  rg (*P*) |
| --- | --- | --- | --- | --- | --- | --- | --- | --- | --- | --- |
|  |  |  |  |  |  |  |  |  |  |  |
| Depressive symptoms | **0.52 (1.60E-46)** | **0.33 (0.0002)** |  | **0.55 (3.41E-30)** | **0.67 (0.00000057)** | **0.48 (1.49E-14)** | 0.34 (0.034) | 0.12 (0.13) |  | **0.69 (1.35E-27)** |
| Anorexia Nervosa | 0.036 (0.25) | 0.062 (0.39) |  | -0.035 (0.41) | 0.25 (0.0027) | 0.047 (0.34) | 0.11 (0.33) | -0.076 (0.31) |  | 0.02 (0.73) |
| Neo-conscientiousness | -0.12 (0.2) | -0.035 (0.87) |  | -0.095 (0.41) | 0.0077 (0.97) | -0.095 (0.5) | -0.07 (0.8) | -0.23 (0.14) |  | -0.12 (0.34) |
| Neo-openness to experience | -0.08 (0.3) | 0.099 (0.59) |  | -0.093 (0.35) | 0.021 (0.91) | -0.31 (0.0097) | -0.15 (0.52) | -0.42 (0.0071) |  | -0.27 (0.032) |
| Neuroticism | **0.5 (2.24E-72)** | **0.3 (0.00001)** |  | **0.44 (2.00E-29)** | **0.7 (2.38E-09)** | **0.4 (1.73E-13)** | 0.27 (0.037) | 0.18 (0.0022) |  | **0.45 (3.42E-17)** |
| Attention deficit hyperactivity disorder | 0.24 (0.0038) | 0.037 (0.82) |  | 0.36 (0.0022) | 0.67 (0.0038) | 0.26 (0.066) | 0.31 (0.22) | 0.42 (0.026) |  | 0.28 (0.047) |
| Bipolar disorder | 0.06 (0.14) | 0.047 (0.63) |  | 0.061 (0.27) | 0.079 (0.46) | -0.02 (0.71) | 0.04 (0.75) | -0.17 (0.037) |  | 0.082 (0.23) |
| PGC cross-disorder analysis | **0.18 (3.05E-06)** | 0.17 (0.11) |  | 0.18 (0.0003) | 0.4 (0.001) | 0.13 (0.034) | 0.23 (0.073) | -0.023 (0.79) |  | 0.24 (0.0004) |
| Major depressive disorder | **0.39 (1.57E-11)** | 0.34 (0.013) |  | **0.4 (5.75E-08)** | 0.53 (0.0005) | **0.36 (0.000034)** | 0.042 (0.8) | -0.071 (0.53) |  | **0.43 (0.0000055)** |
| Autism spectrum disorder | 0.017 (0.75) | 0.046 (0.65) |  | -0.096 (0.078) | 0.027 (0.82) | -0.099 (0.15) | -0.092 (0.54) | 0.078 (0.36) |  | -0.18 (0.032) |
| Schizophrenia | **0.12 (9.08E-06)** | 0.19 (0.0025) |  | 0.098 (0.0045) | 0.22 (0.003) | 0.07 (0.049) | 0.2 (0.036) | -0.014 (0.79) |  | **0.24 (3.33E-08)** |
| Subjective well being | **-0.37 (9.51E-19)** | **-0.35 (0.0001)** |  | **-0.26 (0.0000011)** | **-0.56 (0.000005)** | **-0.33 (0.0000003)** | -0.0013 (0.99) | -0.091 (0.25) |  | **-0.35 ( 1.12E-08)** |
| Neuroticism | **0.52 (1.72E-14)** | 0.48 (0.0029) |  | **0.42 (0.00000053)** | **0.73 (0.0001)** | **0.5 (0.0000021)** | 0.32 (0.13) | 0.2 (0.057) |  | **0.45 (0.000062)** |
| Attention deficit hyperactivity disorder (GC) | 0.34 (0.0026) | 0.21 (0.31) |  | 0.47 (0.0005) | 0.64 (0.029) | 0.37 (0.011) | 0.56 (0.073) | -0.039 (0.82) |  | 0.64 (0.0006) |
| Attention deficit hyperactivity disorder (No GC) | 0.34 (0.0025) | 0.21 (0.31) |  | 0.47 (0.0005) | 0.64 (0.029) | 0.37 (0.011) | 0.56 (0.071) | -0.038 (0.87) |  | 0.64 (0.0006) |

rg: genetic correlation

P < 0.05/ (15 x 8) = 0.00042 was considered as significant.
