# Supplementary material for: Genetic correlations between pain phenotypes and depression and neuroticism
Source: Eur J Hum Genet. 2019 Oct 29;28(3):358–66. doi: 10.1038/s41431-019-0530-2 (PMC7028719; doi:10.1038/s41431-019-0530-2)
Supplement: Supplementary file 3 — Supplementary Table 3 [file 41431_2019_530_MOESM3_ESM.docx]

**Supplementary Table 3.** Mendelian randomisation analysis of the effect of headache on depressive symptoms, neuroticism and major depressive disorders and vice versa

| Outcome | Exposure | Method | Beta | Se | *P* |
| --- | --- | --- | --- | --- | --- |
| Depressive symptoms | Headache | MR Egger | 0.1778 | 0.1925 | 0.3591 |
| Depressive symptoms | Headache | Weighted median | 0.2046 | 0.0724 | **0.0047** |
| Depressive symptoms | Headache | Inverse variance weighted | 0.2243 | 0.0496 | **5.97x10^-06^** |
| Headache | Depressive symptoms | MR Egger | -0.206 | 0.175 | 0.238 |
| Headache | Depressive symptoms | Weighted median | 0.033 | 0.048 | 0.488 |
| Headache | Depressive symptoms | Inverse variance weighted | 0.053 | 0.039 | 0.175 |
| Neuroticism | Headache | MR Egger | 0.1147 | 0.2733 | 0.6761 |
| Neuroticism | Headache | Weighted median | 0.2824 | 0.0751 | **0.0002** |
| Neuroticism | Headache | Inverse variance weighted | 0.3213 | 0.0690 | **3.26x10^-06^** |
| Headache | Neuroticism | MR Egger | 0.882 | 0.570 | 0.122 |
| Headache | Neuroticism | Weighted median | 0.126 | 0.034 | **0.000** |
| Headache | Neuroticism | Inverse variance weighted | 0.142 | 0.049 | **0.004** |
| Major depressive disorder | Headache | MR Egger | 0.3637 | 1.458 | 0.8043 |
| Major depressive disorder | Headache | Weighted median | 0.2855 | 0.4943 | 0.5635 |
| Major depressive disorder | Headache | Inverse variance weighted | 0.2293 | 0.3519 | 0.5147 |
| Headache | Major depressive disorder | MR Egger | 0.007 | 0.021 | 0.744 |
| Headache | Major depressive disorder | Weighted median | 0.005 | 0.007 | 0.501 |
| Headache | Major depressive disorder | Inverse variance weighted | 0.006 | 0.006 | 0.275 |

Significant *P* values (*P* < 0.05) are **in bold**.

The Inverse variance weighted method is the main Mendelian randomisation (MR) method we chose. It consists of a weighted regression of the SNP-outcome regression coefficient on the SNP-exposure regression coefficients constraining the intercept to be zero. (ref 44) MR-egger and Weighted median methods are two sensitivity analyses of the Inverse variance weighted method to check whether the Inverse variance weighted estimates are likely to be biased by unbalanced horizontal pleiotropic effects.

The MR analysis was based on R package Mendelian Randomisation. Only top SNPs with P value < 5 x 10^-8^ were used as genetic instruments.

We used the GWAS data of the depressive symptoms (ref 28), neuroticism phenotype (ref 28), major depressive disorder (ref 30) and headache (ref 42) for the bi-directional MR analysis.
