# Supplementary material for: Genetic correlations between pain phenotypes and depression and neuroticism
Source: Eur J Hum Genet. 2019 Oct 29;28(3):358–66. doi: 10.1038/s41431-019-0530-2 (PMC7028719; doi:10.1038/s41431-019-0530-2)
Supplement: Supplementary file 4 — Supplementary Figure 1 [file 41431_2019_530_MOESM4_ESM.pptx]

## Slide 1
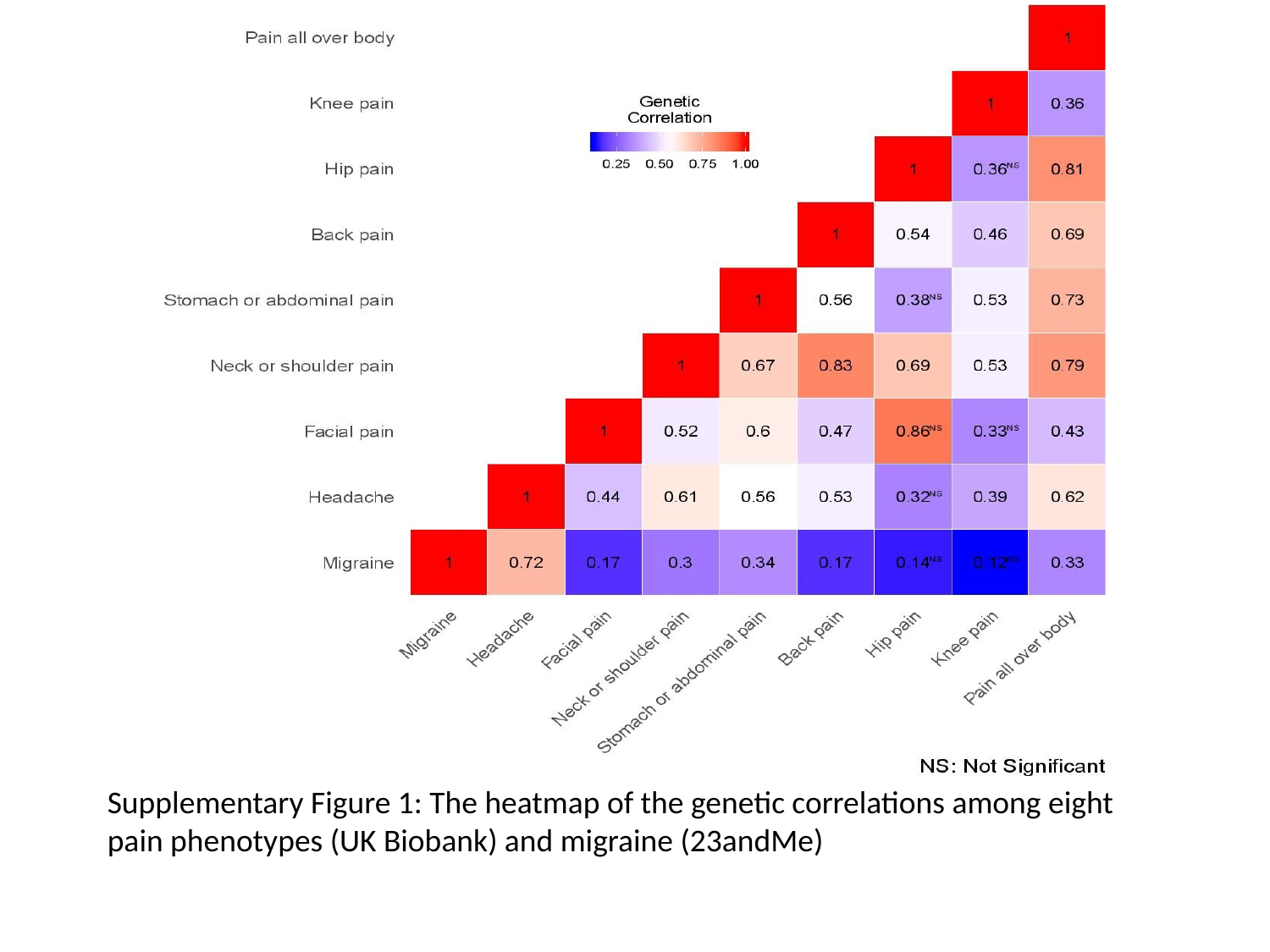

Supplementary Figure 1: The heatmap of the genetic correlations among eight pain phenotypes (UK Biobank) and migraine (23andMe)
