# Supplementary material for: Genetic correlations between pain phenotypes and depression and neuroticism
Source: Eur J Hum Genet. 2019 Oct 29;28(3):358–66. doi: 10.1038/s41431-019-0530-2 (PMC7028719; doi:10.1038/s41431-019-0530-2)
Supplement: Supplementary file 5 — Supplementary file [file 41431_2019_530_MOESM5_ESM.docx]

**Supplementary information**

1. Table: GWAS related information of the eight pain phenotypes defined by the UK Biobank

| Pain phenotypes | Cases | Controls | Lambda |
| --- | --- | --- | --- |
| Headache | 74,761 | 149,312 | 1.31 |
| Facial pain | 2,610 | 149,312 | 1.04 |
| Neck or shoulder pain | 53,994 | 149,312 | 1.20 |
| Stomach or abdominal pain | 8,217 | 149,312 | 1.04 |
| Back pain | 43,991 | 149,312 | 1.13 |
| Hip pain | 10,116 | 149,312 | 1.03 |
| Knee pain | 22,204 | 149,312 | 1.06 |
| Pain all over body | 5,670 | 149,312 | 1.09 |

UK Biobank Pain Question

‘In the last month have you experienced any of the following that interfered with your usual activities?’. The options were: 1. Headache; 2. Facial pain; 3. Neck or shoulder pain; 4. Back pain; 5. Stomach or abdominal pain; 6. Hip pain; 7. Knee pain; 8. Pain all over the body; 9. None of the above; 10. Prefer not to say. More than one option could be selected. (UK Biobank Questionnaire field ID: 6159)

For each pain phenotype, cases were defined as those who selected the specific pain site option for the above question, regardless of whether they had selected other options. For example, headache cases are those who selected the ‘Headache’ option; Facial pain cases are those who selected the ‘Facial pain’ option; etc.

For each GWAS analysis, controls were those who selected the ‘None of the above’ option. Thus we used the same ‘no pain’ control population for all pain phenotypes in different body sites.

1. Manhattan plots and Q-Q plots of eight pain phenotypes defined by the UK Biobank:
   1. Pain all over the body
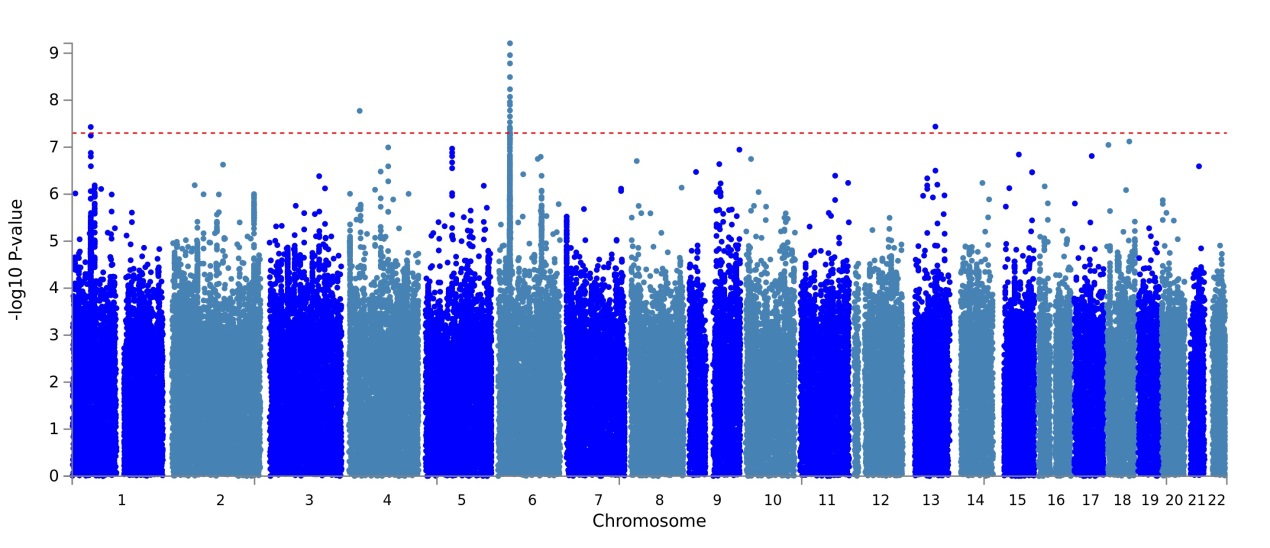

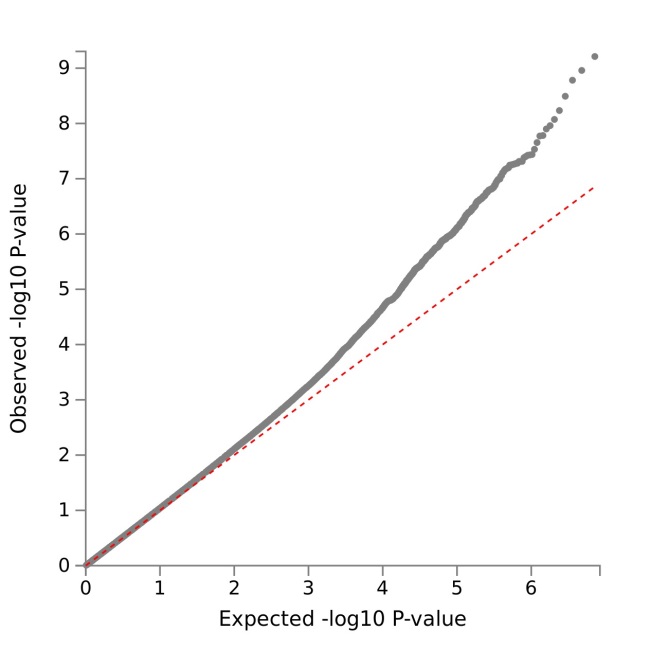


Lambda: 1.09

- 1. Knee pain


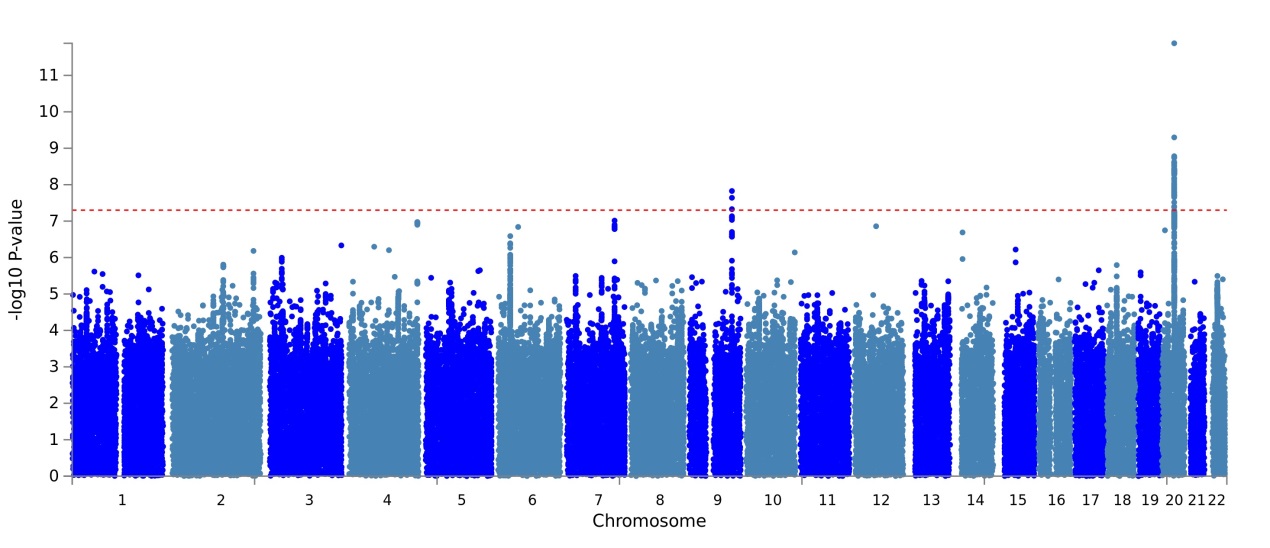


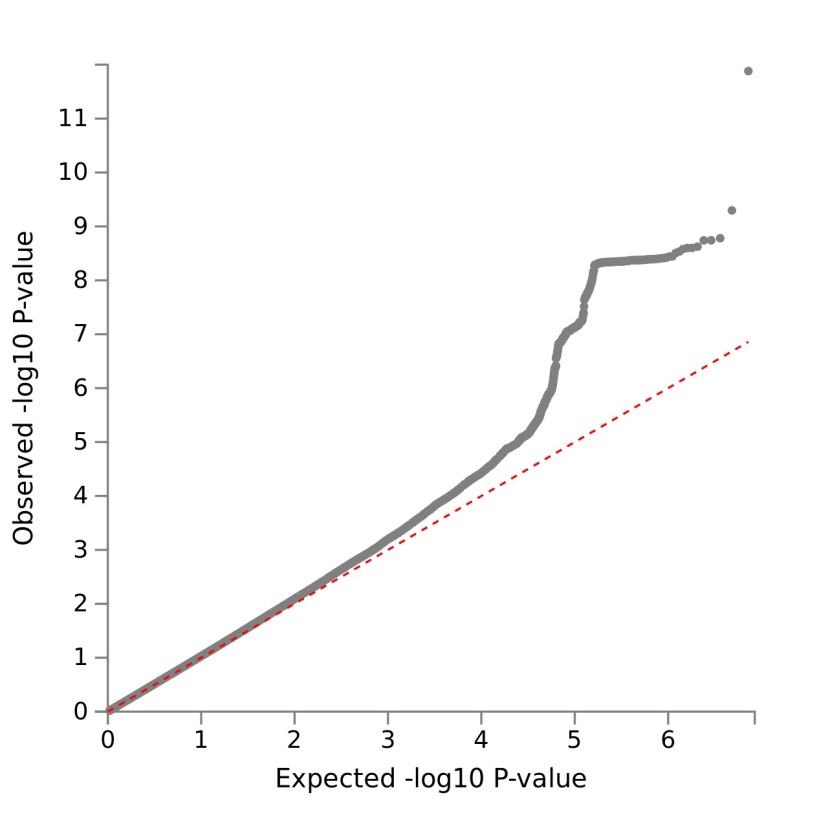


Lambda: 1.06

- 1. Neck and shoulder pain


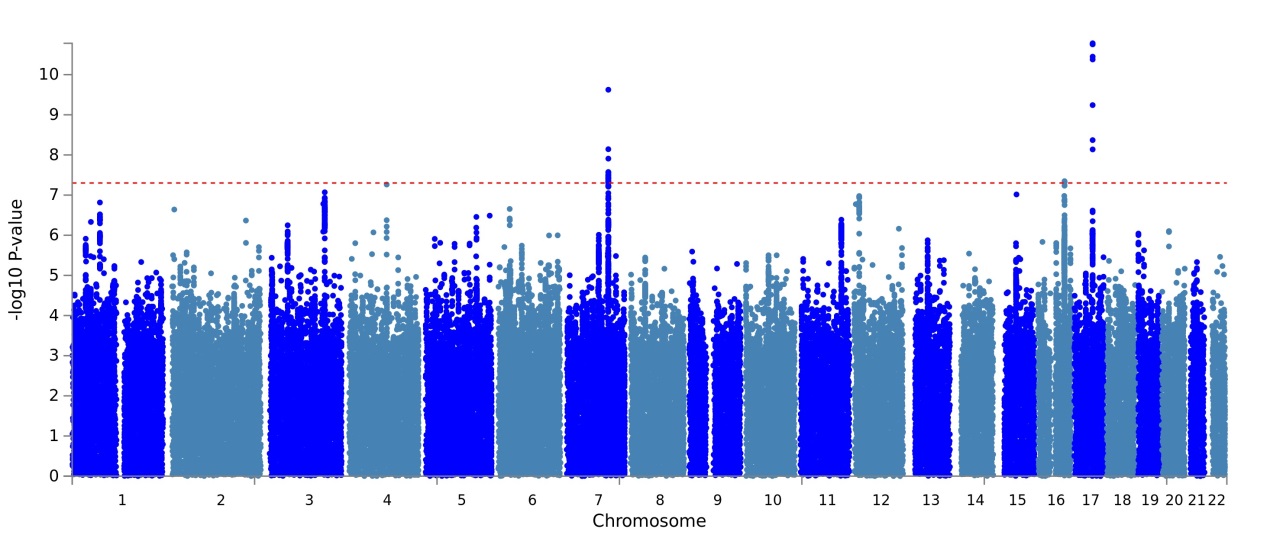


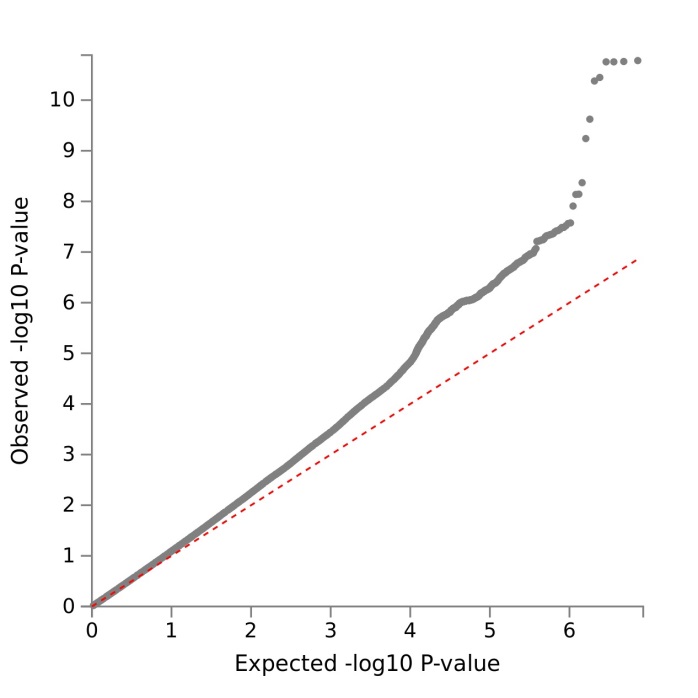


Lambda: 1.20

- 1. Abdominal pain


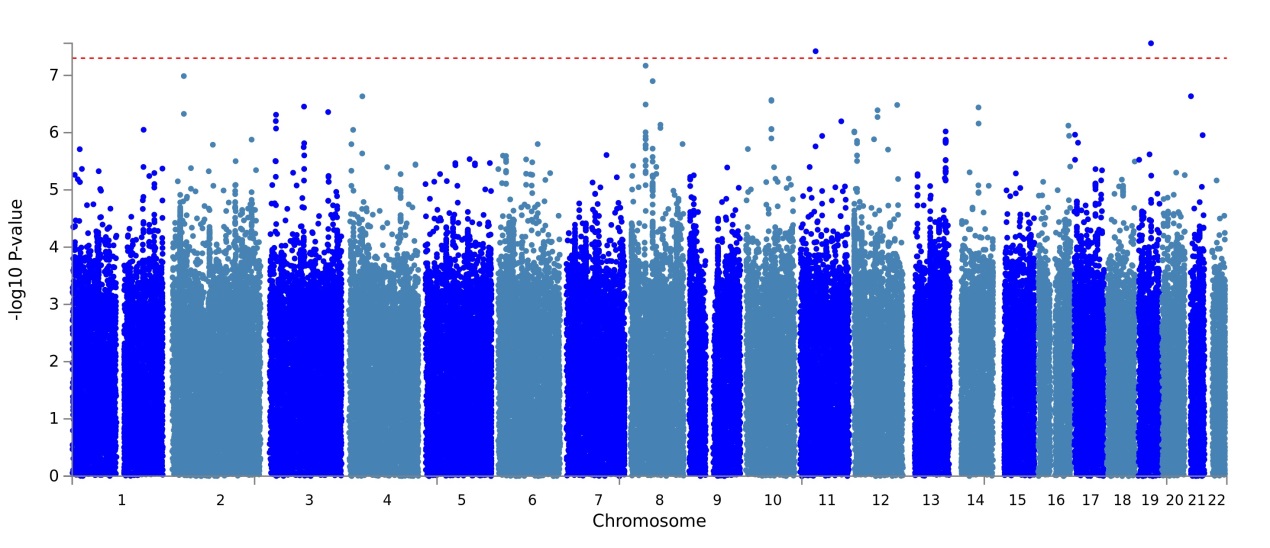


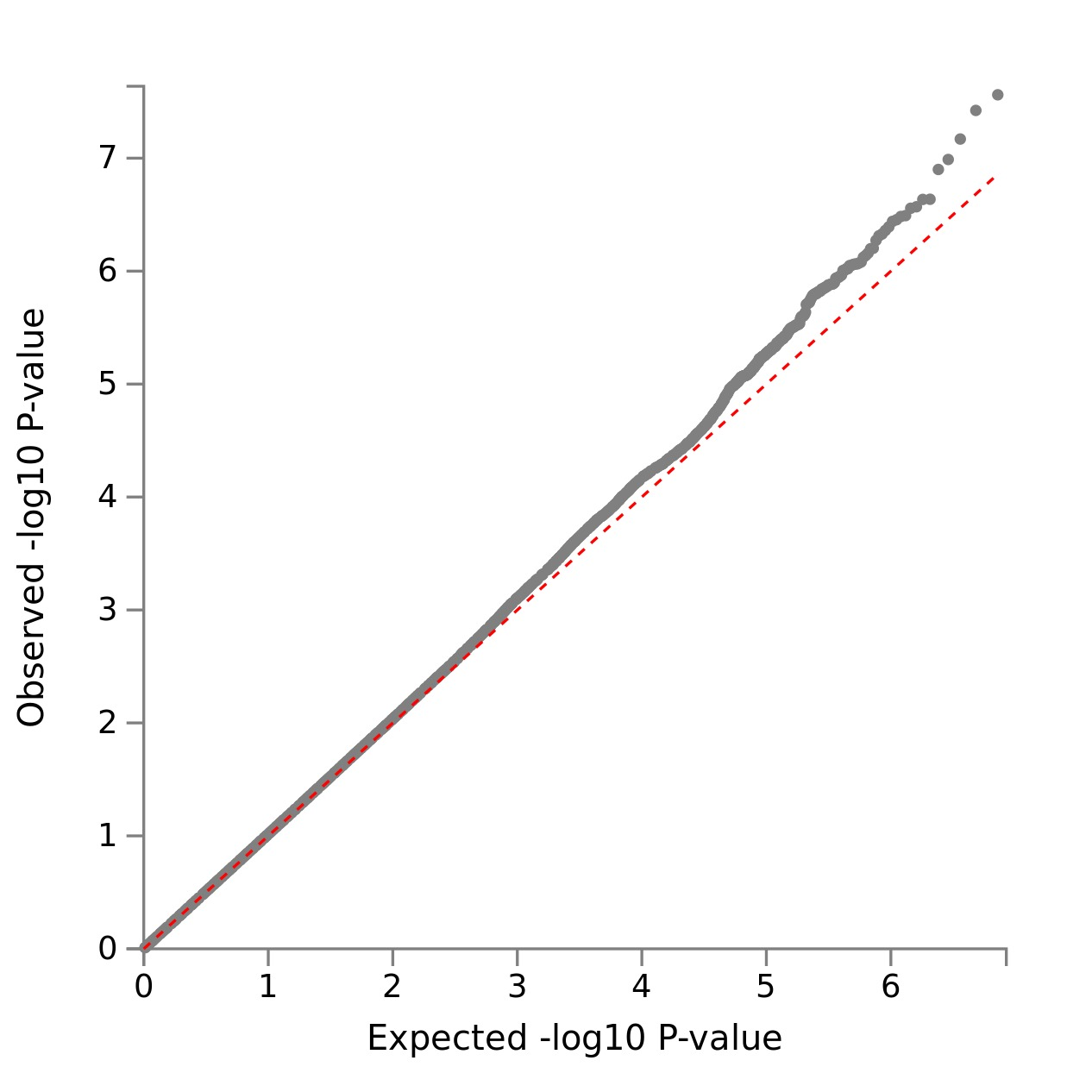


Lambda: 1.04

- 1. Hip pain


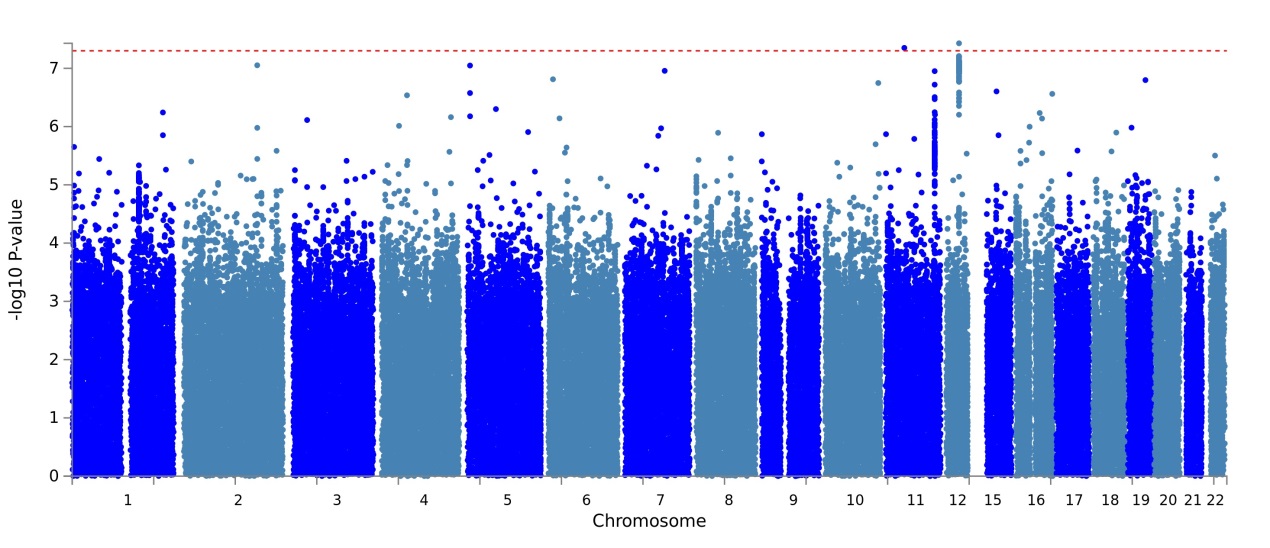


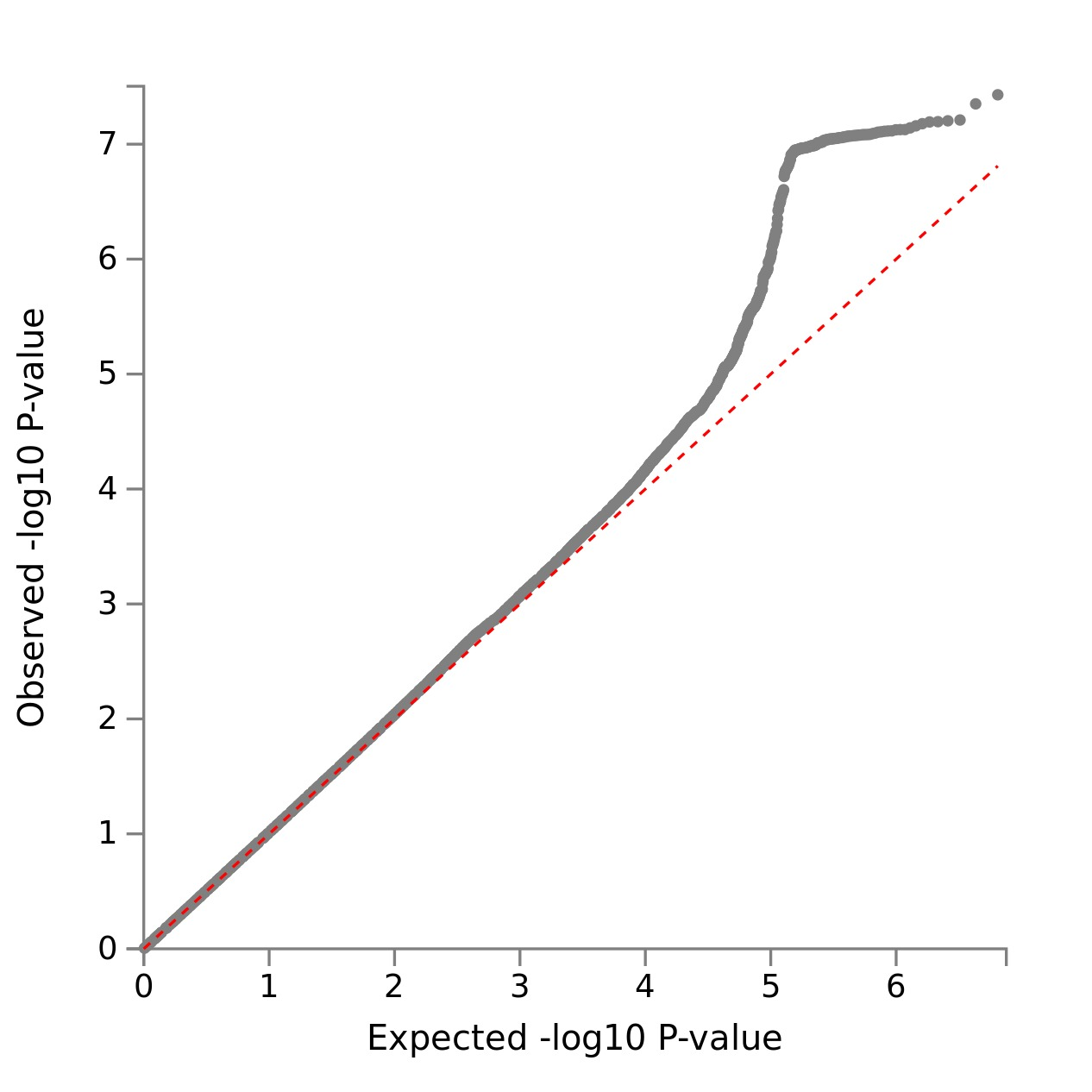


Lambda: 1.03

- 1. Back pain


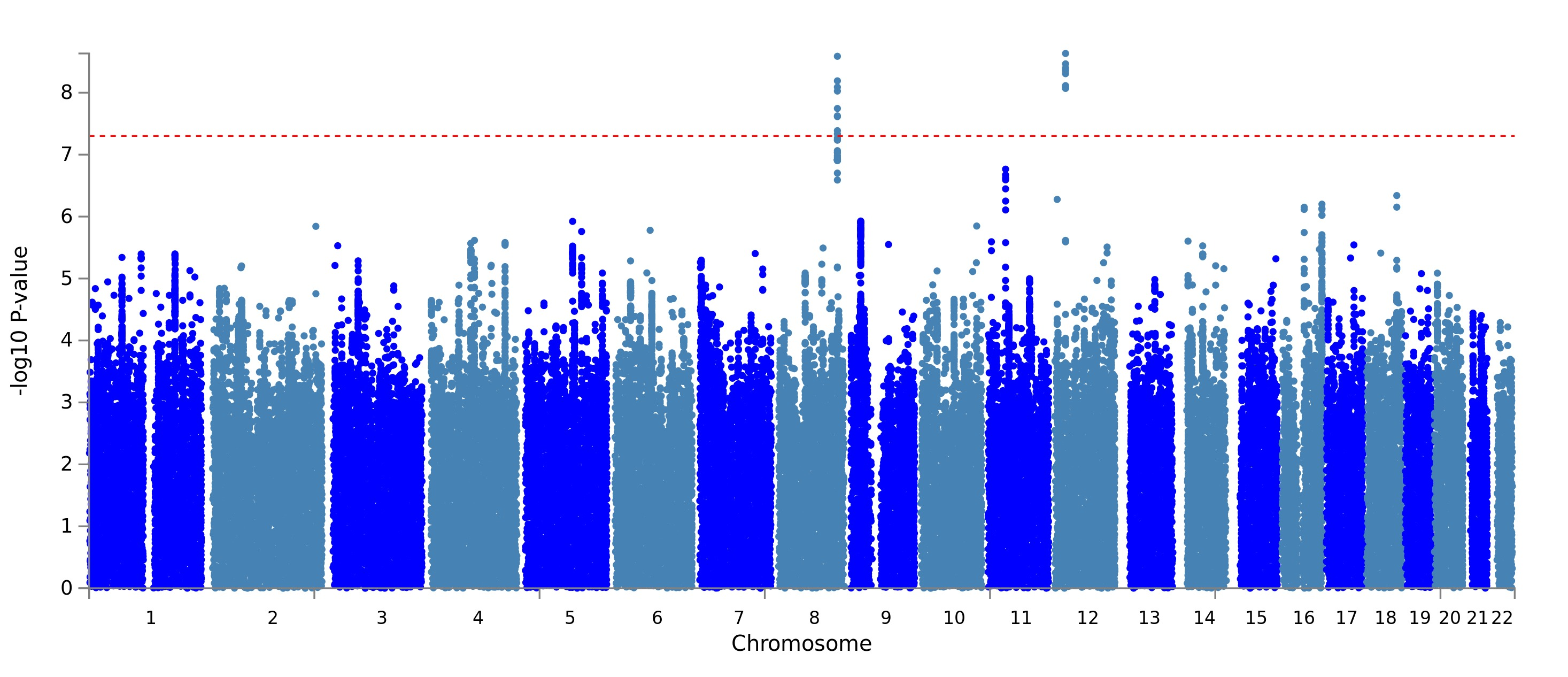


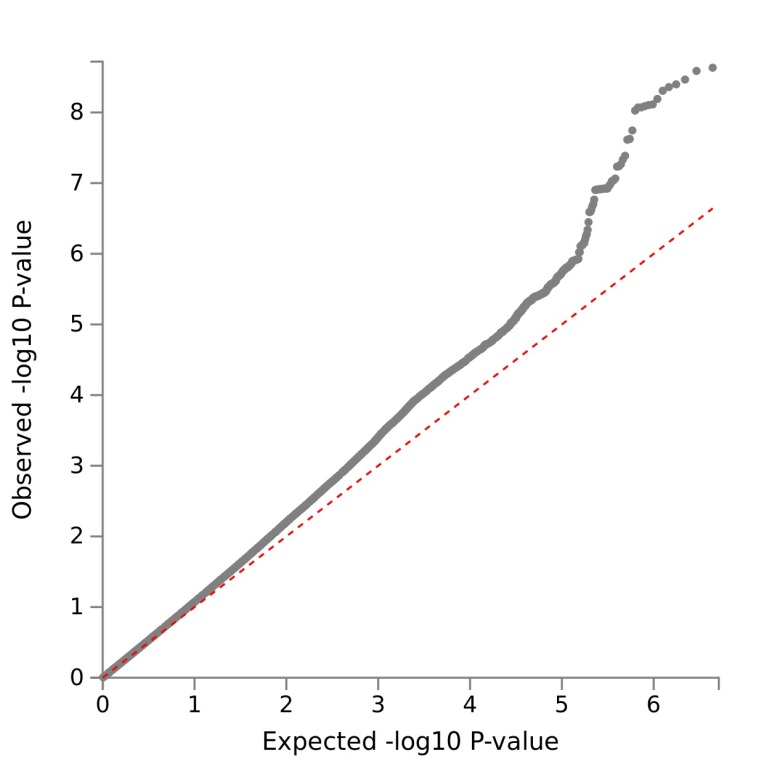


Lambda: 1.13

- 1. Facial pain


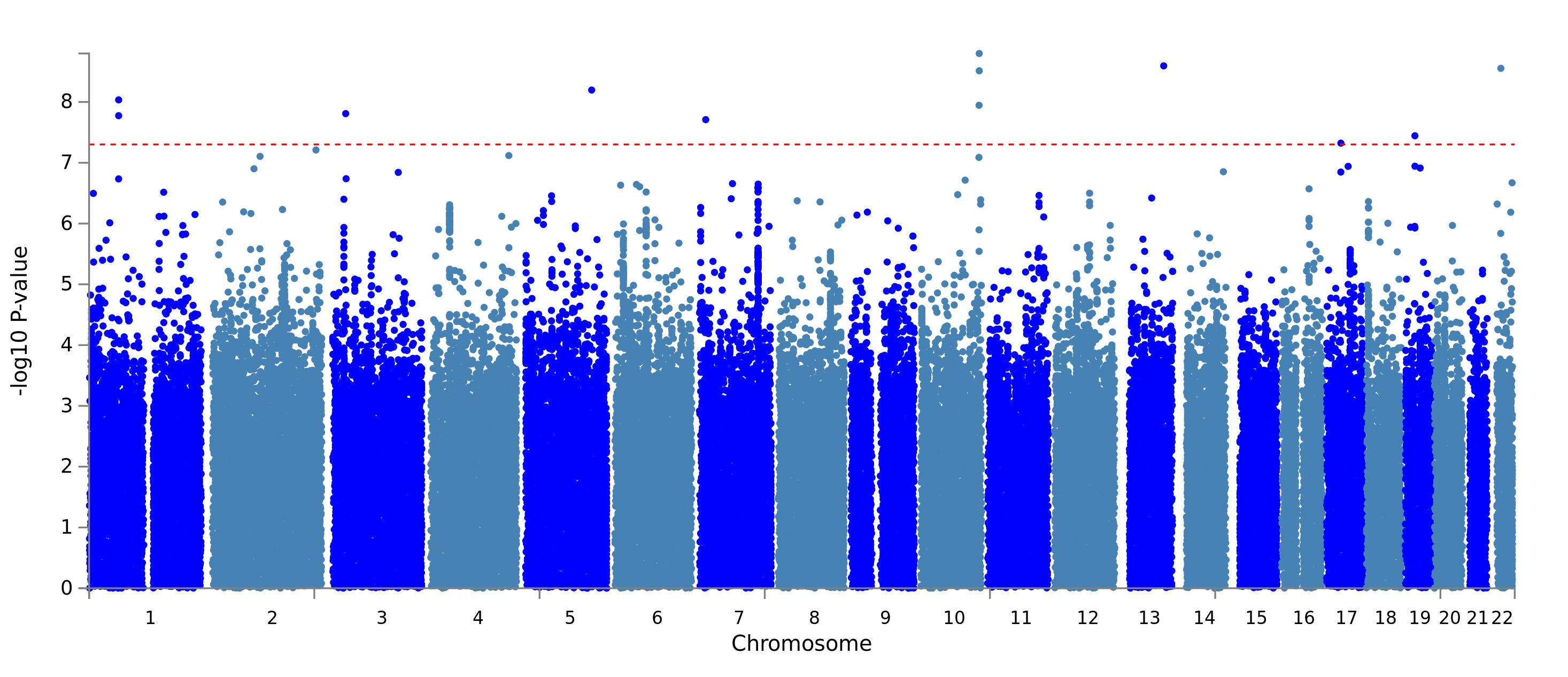


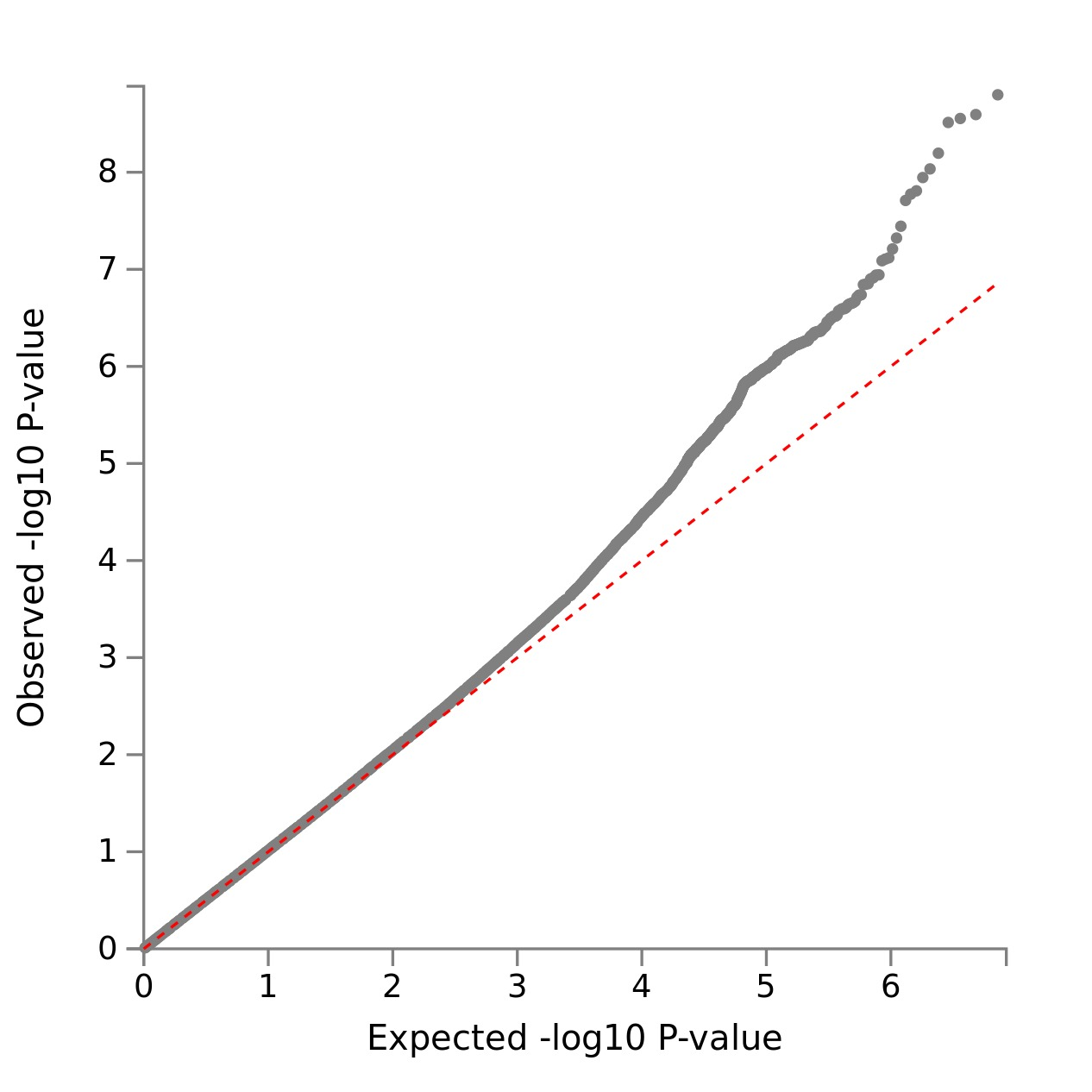


Lambda: 1.04

- 1. Headache


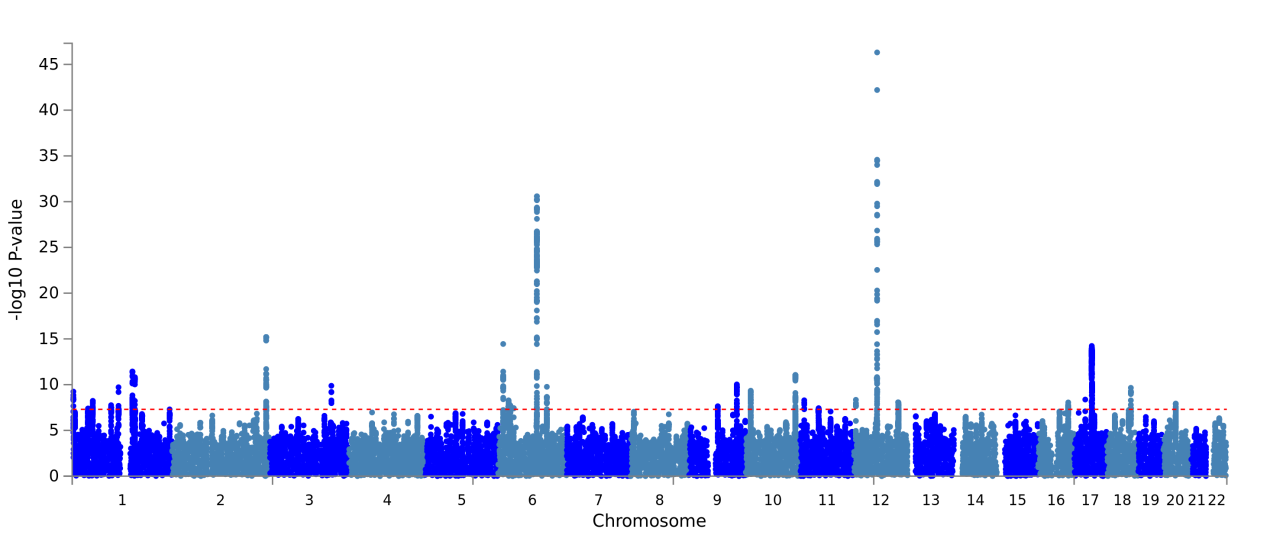


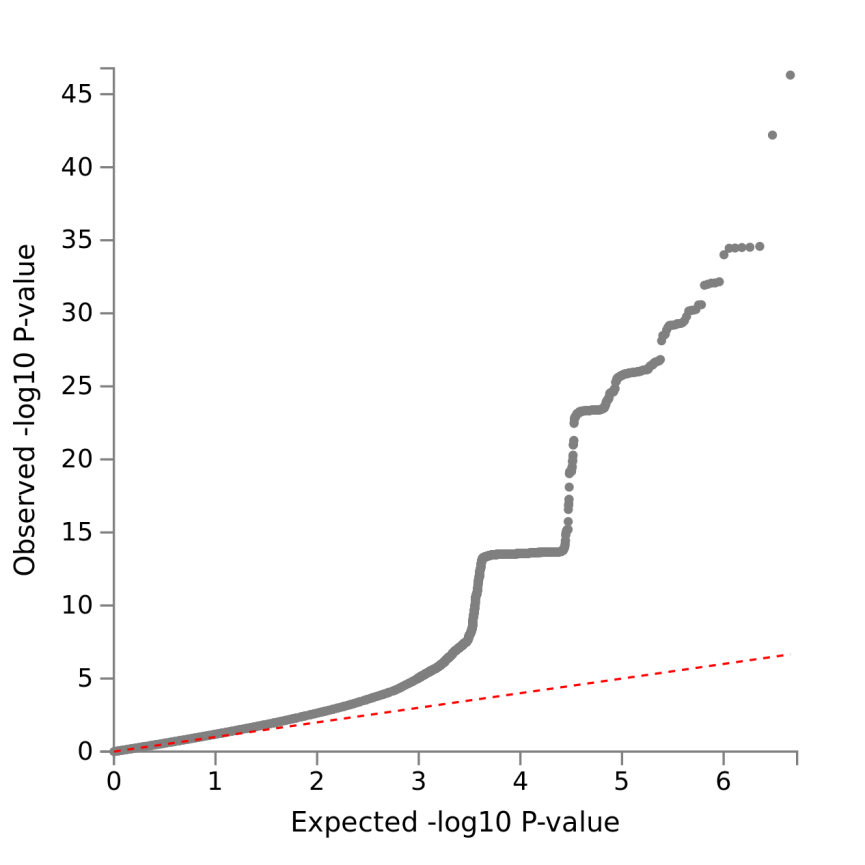


Lambda: 1.31

Please note, the headache GWAS has been published.

https://www.ncbi.nlm.nih.gov/pubmed/29397368
